# Relationship between Nanomechanical Responses of Interfacial Intermetallic Compound Layers and Impact Reliability of Solder Joints

**DOI:** 10.3390/nano10081456

**Published:** 2020-07-25

**Authors:** Jenn-Ming Song, Bo-Chang Huang, David Tarng, Chih-Pin Hung, Kiyokazu Yasuda

**Affiliations:** 1 Department of Materials Science and Engineering, National Chung Hsing University, Taichung 402, Taiwan; 2 Division of Materials and Manufacturing Science, Graduate School of Engineering, Osaka University, Osaka 565-0817, Japan; yasuda@mapse.eng.osaka-u.ac.jp; 3 Research Center for Sustainable Energy and Nanotechnology, National Chung Hsing University, Taichung 402, Taiwan; 4 Innovation and Development Center of Sustainable Agriculture, National Chung Hsing University, Taichung 402, Taiwan; 5 Department of Materials Science and Engineering, National Dong Hwa University, Hualien 974, Taiwan; m9822009@ems.ndhu.edu.tw; 6 Advanced Semiconductor Engineering Group, Kaohsiung 811, Taiwan; David_Tarng@aseglobal.com (D.T.); cp_hung@aseglobal.com (C.-P.H.)

**Keywords:** solder joint, intermetallic compounds, nanoindentation, impact test

## Abstract

This study aims to evaluate solder joint reliability under high speed impact tests using nanoindentation properties of intermetallic compounds (IMCs) at the joint interface. Sn–Ag based solder joints with different kinds of interfacial IMCs were obtained through the design of solder alloy/substrate material combinations. Nanoindentation was applied to investigate the mechanical properties of IMCs, including hardness, Young’s modulus, work hardening exponent, yield strength, and plastic ability. Experimental results suggest that nanoindentation responses of IMCs at joint interface definitely dominates joint impact performance. The greater the plastic ability the interfacial IMC exhibits, the superior impact energy the solder joints possess. The concept of mechanical and geometrical discontinuities was also proposed to explain brittle fracture of the solder joints with bi-layer interfacial IMCs subject to impact load.

## 1. Introduction

With portable devices developing rapidly towards multi-function and miniaturization, the reliability of the board-to-package joints has become more important. The joints between ball grid array (BGA) packages and the printed circuit boards are usually assembled without the support of underfills. When the electronics fall to the ground, the board-to-package joints are subject to impact load and then causes damages readily. In order to study the board-level solder joint reliability, a ball impact test (BIT) suffering high strain rate deformation was developed. Similar to a board-level drop test, BIT usually gives rise to brittle fracture at the interfacial IMCs [1,2].

**Figure 1:**
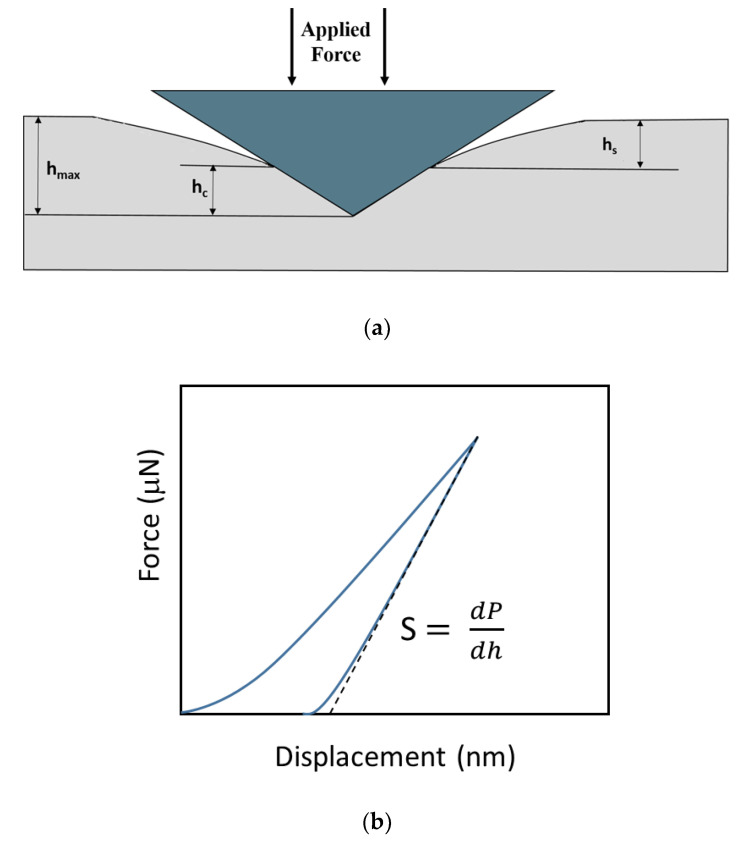
Illustration of nanoindentation test: (**a**) indentation profile at maximum load, and (**b**) typical load-displacement curves.

Since the device failure caused by solder joint damages usually arises from IMCs cracking when subjected to drop test, the mechanical features of IMCs significantly influence joint reliability. Accordingly, nanoindentation has been used to investigate the mechanical properties of interfacial IMCs of solder joints because of the small area [3,4,5,6,7]. As illustrated in Figure 1, nanoindentation is a depth sensing indentation technique, for which the load and depth that the indenter penetrates into the samples surface are simultaneously recorded. The hardness is defined by [8]

$$H=\frac{P_{max}}{A_{c}}$$

where *P_max_* stands for the maximum indentation load applied during testing, and *A_c_* stands for the projected contact area which could be obtained by measuring the depth of indenter penetration into the specimen surface as well as the indenter geometry. The reduced elastic modulus is obtained using [8]

$$E_{r}=\frac{S}{2\beta }\sqrt{\frac{\pi }{A_{c}}}$$

where *β* is the shape constant of the Berkovich indenter tip and *S* is the slope of elastic unloading, as shown by *dP/dh* in Figure 1b. Assuming both the sample and indenter are experienced in elastic deformation, we have [8]

$$\frac{1}{E_{r}}=\left(\frac{1-v_{t}{}^{2}}{E_{t}}+\frac{1-v_{i}{}^{2}}{E_{i}}\right)$$

where *E_r_* and *v* stand for reduced modulus and Poisson’s ratio, respectively, while subscripts *t* and *i* indicate sample and indenter. In addition to hardness and elastic modulus, nanoindentation can also be applied to estimate fracture toughness for brittle materials [8]. Mechanical behavior related to deformation rate including strain rate sensitivity and creep can also be derived.

Numerous studies have been devoted to the mechanical behavior of IMCs in solder joints. Effects of alloying, growth texture, allotropic transition, crystal structure and compositions of IMCS were well studied [7,9,10,11,12,13]. However, the relationships between IMC mechanical properties and solder joint reliability have not been systematically investigated yet. Our previous reports suggested that the plastic ability can be evaluated by ratio of elastic modulus/hardness (E/H) [9,11,12]. High E/H indicates better plastic ability for IMCs, while low E/H implies brittleness.

To clarify the role of interfacial IMCs on the BIT reliability from the aspect of mechanical behavior, Sn–Ag based solder joints with different common metallic substrates (Cu, Ag and Ni) were prepared. BIT results of solder joints and nanoindentation responses of various interfacial IMCS were studied and correlated. The strategies to improve impact reliability of soldered packages through the design of nanoindentation properties for interfacial IMCs were proposed.

## 2. Experimental Procedures

760-μm diameter Sn–Ag based solder balls with the compositions of Sn-3.0wt%Ag, Sn-3.5wt%Ag-0.5wt%Cu, Sn-3.0wt%Ag-1.5wt%Zn were designated as SAC, SA and SAZ, respectively. FR4 printed circuit boards with Cu, Ni or Ag pads defined by solder masks were employed. The reflow process was carried out in an IR furnace under a 5%H_2_ + 95%N_2_ reductive atmosphere. The peak temperature was set at 250 °C and the dwell time was 5 s.

A scanning electron microscope (SEM, JEOL JSM-6460, JOEL Ltd., Tokyo, Japan) with electron backscatter diffraction (EBSD, Oxford/HKL, Oxford Instruments, London, UK) was applied to observe the cross-sectional microstructure of solder joints. EBSD was applied for phase identification. EBSD principle and practice are given in detail elsewhere [14]. The EBSD data noise was reduced using the “Tango” module of HKL CHANNEL5 software (Oxford Instruments, London, UK).

The mechanical properties of the IMC phases were determined using nanoindentation. A Nanoindenter XP (MTS, Eden Prairie, MN, USA) with a continuous stiffness measurement module was used. The indentation process approached with a constant strain rate of 5 × 10^−2^ s^−1^, and the loading proceeded up to a total displacement of 300 nm. The measured load-displacement data were interpreted using the method developed by Oliver and Pharr [15]. The hardness and Young’s modulus as a function of the indenter displacement could be obtained. Each datum was the average of ten tests.

An Instron micro-impact test apparatus was applied for BIT testing. The principle and practice of BIT are described in detail elsewhere [1,2,16,17,18]. A striker was ejected upon the release of an air-compressed spring of which the velocity (*V_i_*) was 0.6 m/s in this study. The impact force against time duration was recorded. Peak impact force (*F_max_*) and the impact energy applied during the ascending portion (*E_i_*), which represents the toughness of the solder joints, were measured. A positive relationship has been demonstrated between board-level package drop life and impact energy of solder joints [19]. Thereby, the impact energy determined from BIT tests can be seen as an indication for board-level reliability, subject to drop tests.

## 3. Results and Discussion

**Figure 2:**
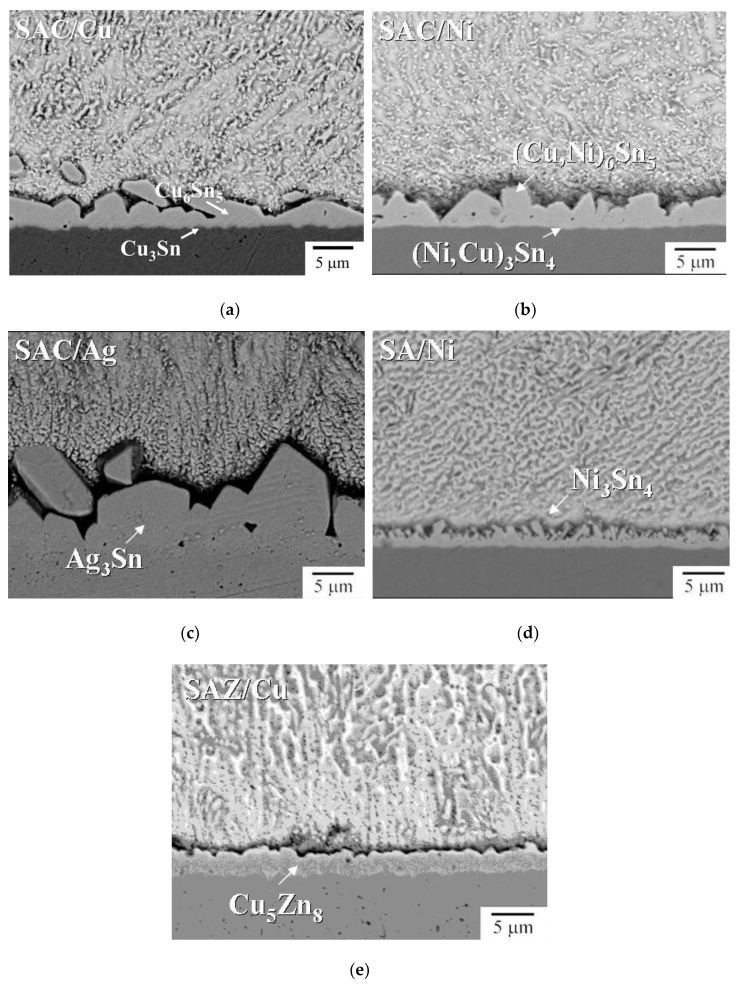
Cross-sectional microstructure of joints: (**a**) SAC/Cu, (**b**) SAC/Ni, (**c**) SAC/Ag, (**d**) SA/Ni and (**e**) SAZ/Cu (the lower side was substrate, while the upper side was solder).

**Figure 3:**
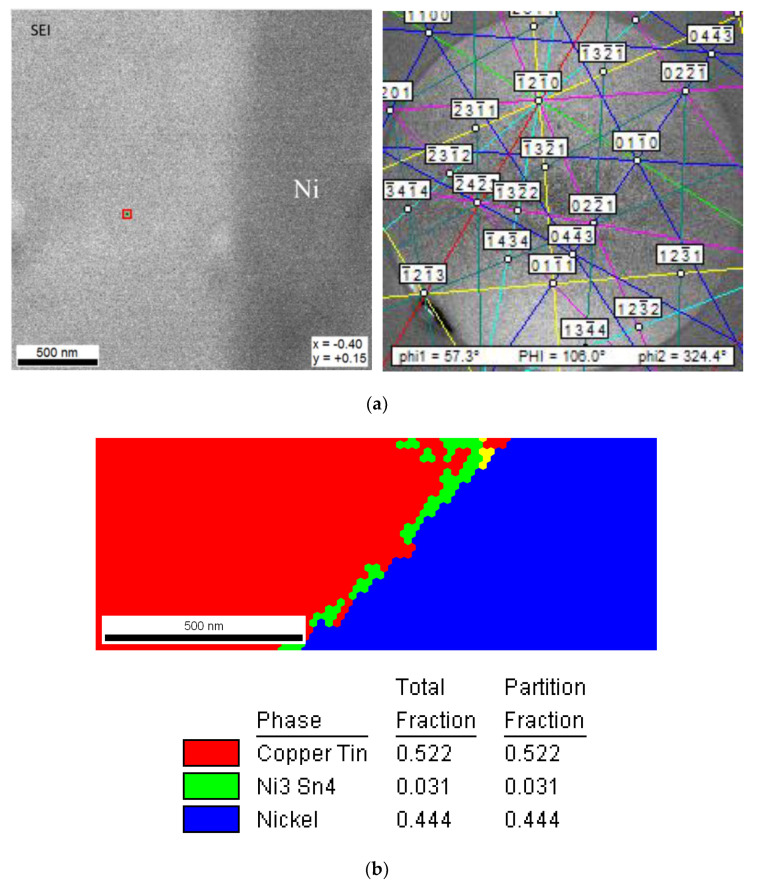
EBSD analytic results of interfacial IMCs at SAC/Ni: (**a**) the spot in the vicinity of Ni substrate and corresponding electron backscatter diffraction pattern, and (**b**) phase mapping.

**Figure 4:**
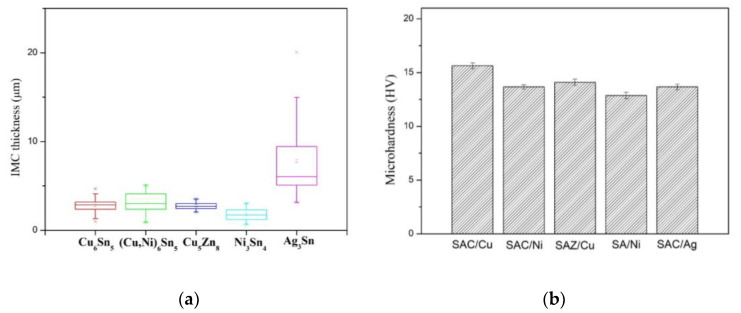
Solder joint properties: (**a**) thickness of interfacial IMCs and (**b**) microhardness of the solder portion.

Figure 2a–e show the interfacial morphology of the joints. Based on the compositional analytical results in Table 1, the interfacial IMCs at joint interface could be identified. As indicated in Figure 2a,b, SAC/Cu and SAC/Ni joints both show bi-layer IMC structure at interface. As for SAC/Cu, the scallop-like IMC next to the solder was Cu_6_Sn_5_, while the thin gray layer adjacent to the substrate with the thickness of 0.5 μm was supposed to be Cu_3_Sn [3]. As for SAC/Ni, the IMC next to the solder was (Cu,Ni)_6_Sn_5_, but the thin bright layer underneath was too thin to detect. Figure 3a illustrates ESBD results obtained from the spot 500 nm from the Ni substrate. The corresponding electron backscatter diffraction pattern and phase mapping, as shown in Figure 3b, verify the structure of Ni_3_Sn_4_. According to the literature, this thin layer would be (Ni,Cu)_3_Sn_4_ [20,21]. The other three samples all reveal single layer feature. They were, Ag_3_Sn at SAC/Ag, Ni_3_Sn_4_ at SA/Ni and Cu_5_Zn_8_ at SAZ/Cu. The quantitative data of interfacial IMC layer thickness are given in Figure 4a, indicating that, except for the extraordinarily thick Ag_3_Sn, the average IMC thickness ranged from 2 to 4 μm. Considering the solder portion of the joints, the microhardness values given in Figure 4b did not differ too much, ranging between 12.5 and 15.5 Hv.

**Figure 5:**
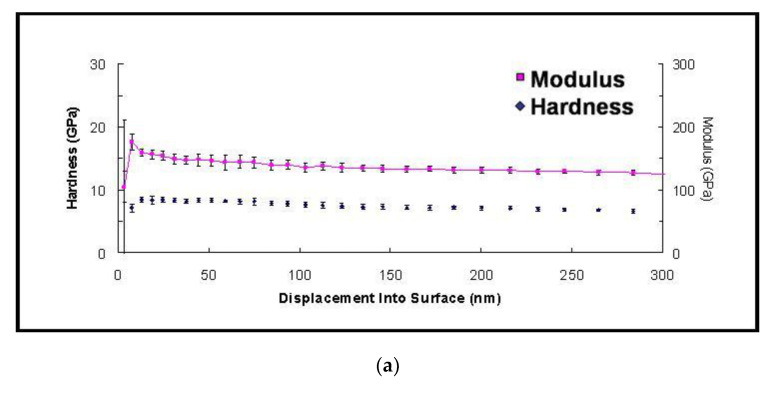

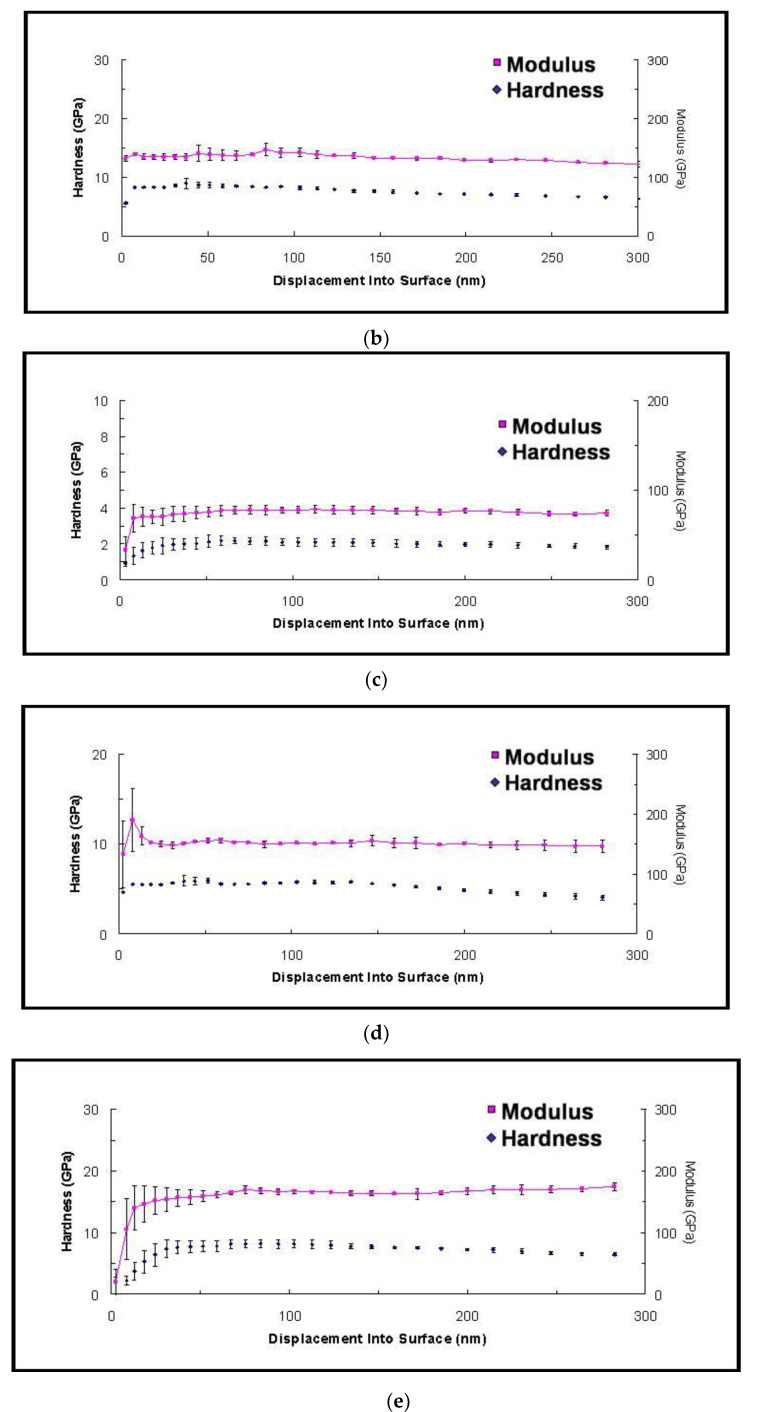
Variation in hardness and elastic modulus of intermetallic compounds along the penetration depth into sample surface: (**a**) Cu_6_Sn_5_, (**b**) (Cu,Ni)_6_Sn_5_, (**c**) Ag_3_Sn, (**d**) Ni_3_Sn_4_ and (**e**) Cu_5_Zn_8_.

To investigate the mechanical properties of interfacial IMCs, nanoindentation tests were made perpendicular to the cross-sectional surface of each sample. The indenter penetrated into the sample up to 300 nm, and the hardness, as well as Young’s modulus, were continuously recorded. Representative charts are displayed in Figure 5. Following an initial transient stage, the measured hardness and Young’s modulus during the indentation remained almost unchanged, indicating the homogeneity of the structural phase. In order to obtain stable data and suppress the substrate effect, the hardness and Young’s modulus within the penetration depth from 200 nm to 250 nm were adopted.

The average hardness and elastic modulus thus obtained are listed in Table 2. It was found that Cu-based IMCs (Cu_6_Sn_5_, (CuNi)_6_Sn_5_ and Cu_5_Zn_8_) possessed higher hardness and elastic moduli; however, their E/H values were lower than the others. This implies Cu-based IMCs possessed excellent plastic deformation resistance but poor plastic ability. Compared with Cu_6_Sn_5_ and (CuNi)_6_Sn_5_, Cu_5_Zn_8_ exhibited greater elastic modulus and E/H. Ag_3_Sn, which exhibited the lowest hardness and elastic modulus, showed superior E/H value.

**Figure 6:**
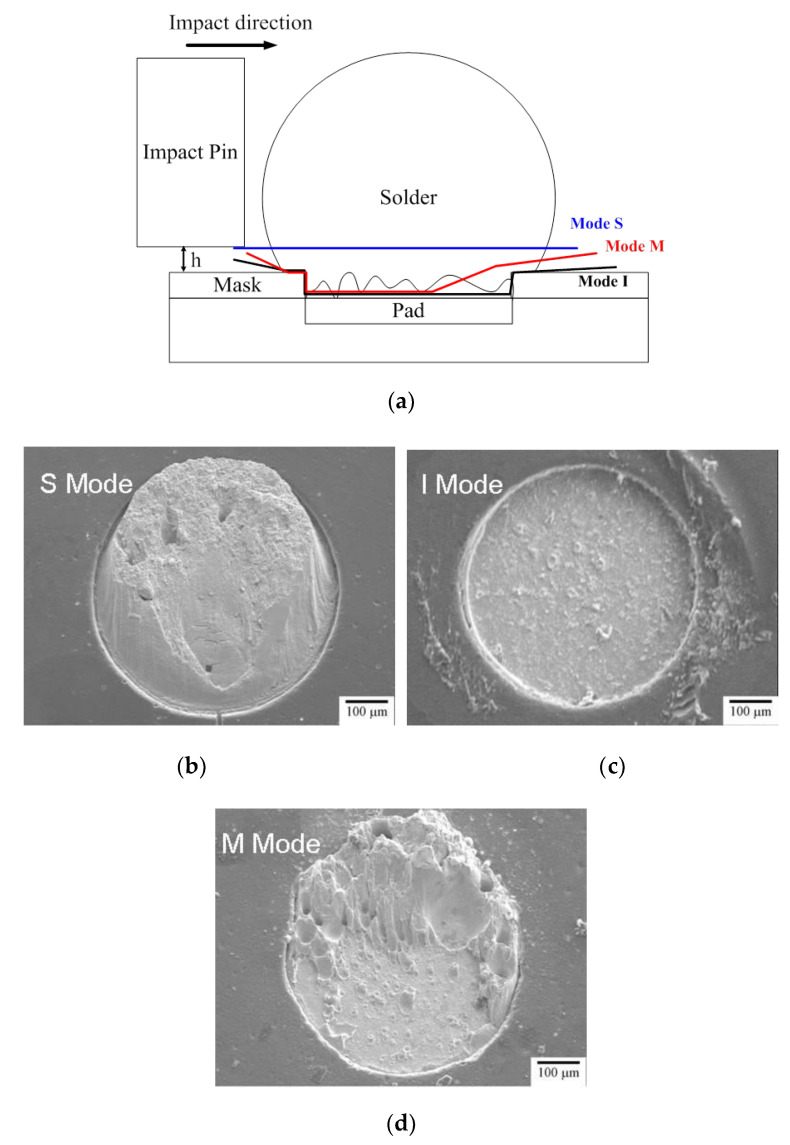
(**a**) Fracture paths of ball-impact-tested solder joints and the definition of fracture modes, (**b**) the S mode with ductile fracture, (**c**) the I mode with brittle fracture along the interface, and (**d**) the M mode with the mixture of S and I modes.

**Figure 7:**
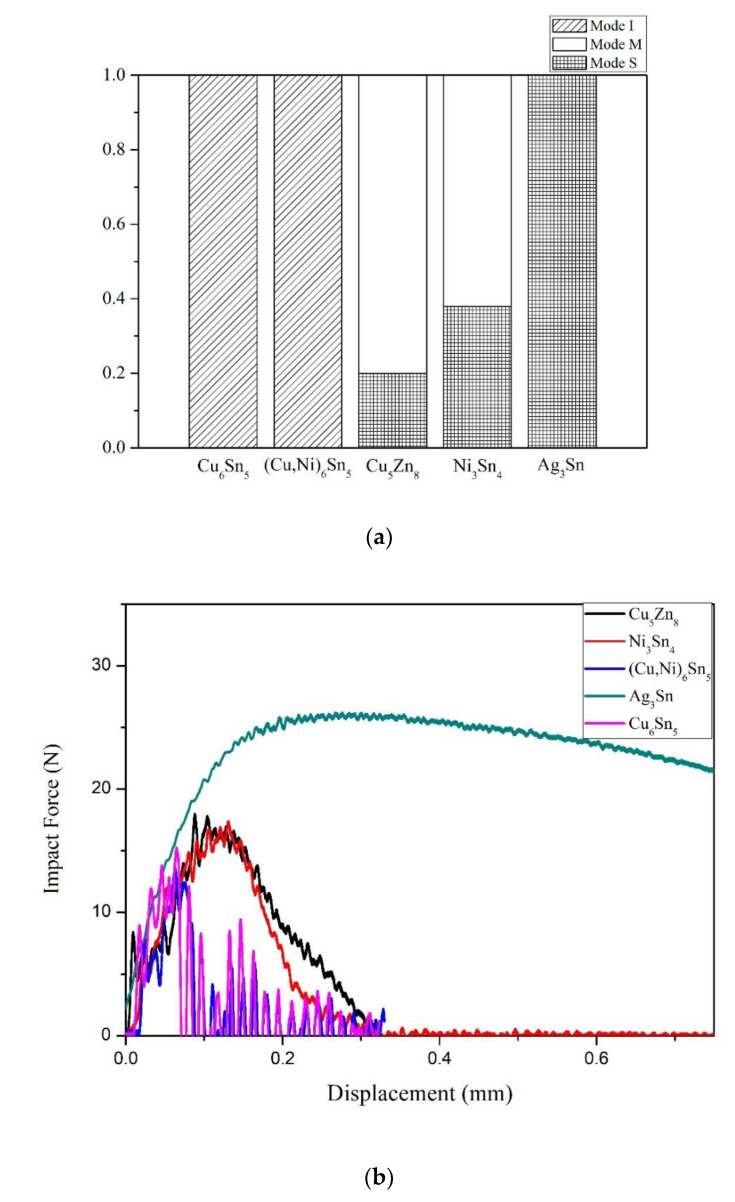
(**a**) The proportions of the joint samples with different fracture modes, and (**b**) corresponding force–displacement curves of the solder joints during impact test.

Figure 6 shows the failure paths of the joints subject to impact loads. As indicated, fracture modes can be divided into three types based on the cracking paths, as shown in Figure 6a. They are the S mode for which the crack propagates inside the solder revealing ductile failure, as shown in Figure 6b, the I mode with cracking all along joint interface, as shown in Figure 6c, and the M mode, with crack propagation beginning from the interface and ending within the solder, as shown in Figure 6d. The proportions of the joint samples with different fracture modes are given in Figure 7a, indicating that all the SAC/Ag joints showed S mode fracture, while SA/Ni and SAZ/Cu exhibited major M mode fracture feature with minor S mode. It is notable that all the joints with bi-layer interfacial IMCs, SAC/Ni and SAC/Cu, revealed brittle I mode fracture characteristics. If the impact force–displacement curves, as shown in Figure 7b, are correlated with above fracture mode features, the area under the impact force–displacement profiles, usually regarded as impact toughness, in the decreasing order, was S mode (SAC/Ag), M mode (SAZ/Cu and SA/Ni) and then I mode (SAC/Cu and SAC/Ni).

**Figure 8:**
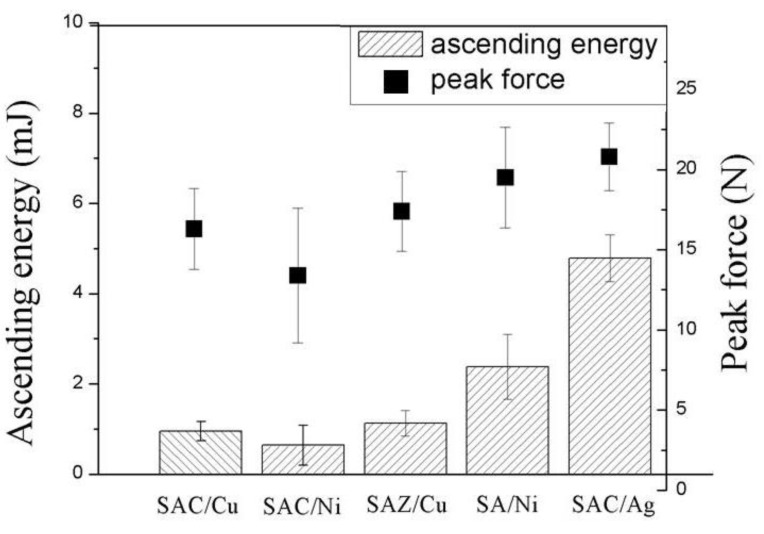
Average impact energy and peak impact force of the solder joints.

In combination of measured peak force and impact energy, Figure 8 concludes that the joints showing the S fracture mode exhibited excellent impact fracture toughness as well as impact force ((SAC/Ag), while those revealing brittle I fracture mode behaved worse (SAC/Cu and SAC/Ni). The performances of the joints with major mixed fracture mode (M mode; i.e., SAZ/Cu and SA/Ni) were in the middle of them.

**Figure 9:**
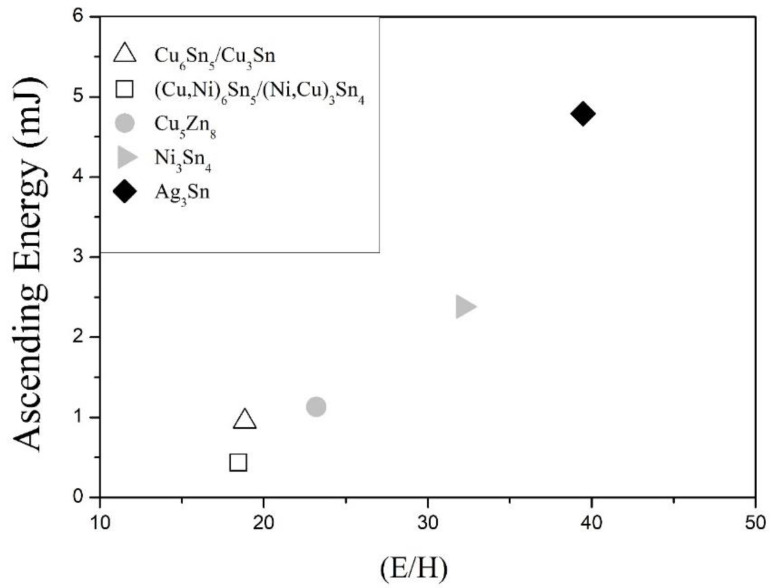
Impact energy of the solder joints against plastic ability (E/H) of interfacial IMCs.

It is interesting that, from the view-point of IMCs’ nanoindentation responses, the joint impact performance can be perfectly related to plastic ability of interfacial IMCs, as shown in Figure 9. As illustrated, the joint impact energy was directly proportional to E/H value. This suggests that the mechanical behavior of IMCs at joint interfaces definitely dominates joint reliability under high deformation rate conditions.

**Figure 10:**
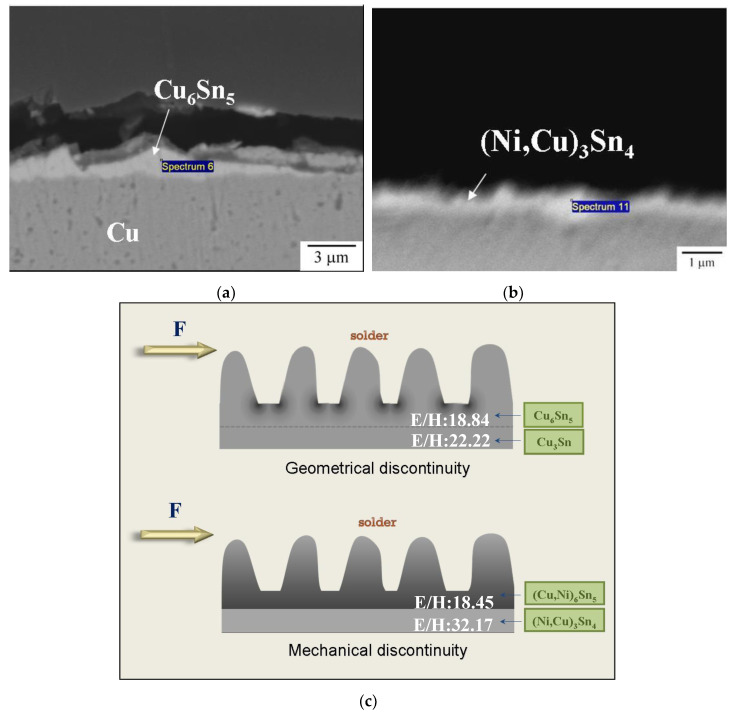
Cross-section of the fractured samples with two interfacial IMC layers: (**a**) Cu_6_Sn_5_/Cu_3_Sn and (**b**) (Cu,Ni)_6_Sn_5_/Ni_3_Sn_4_, and (**c**) the proposed mechanism affecting crack propagation path.

As also shown in Figure 9, the joints with bi-layered interfacial IMCs possessed poor joint impact performance and completely brittle fracture feature. (Cu,Ni)_6_Sn_5_/(Ni,Cu)_3_Sn_4_ was even worse than Cu_6_Sn_5_/Cu_3_Sn. This could be explained by the E/H difference between two interfacial IMC layers. The magnified cross-sectional images given in Figure 10a,b indicate that, for Cu_6_Sn_5_/Cu_3_Sn, the main crack propagated mostly within the upper Cu_6_Sn_5_, while as to (Cu,Ni)_6_Sn_5_/(Ni,Cu)_3_Sn_4_, the crack passed along the interface between (Cu,Ni)_6_Sn_5_ and (Ni,Cu)_3_Sn_4_. This inconsistency could be referred to geometrical discontinuity and mechanical discontinuity of the bi-layer IMCs, sketched in Figure 10c. With respect to Cu_6_Sn_5_/Cu_3_Sn, the E/H values for Cu_6_Sn_5_ (18.84) and Cu_3_Sn (22.22) [12] did not diverge too much, therefore the crack propagated within Cu_6_Sn_5_, which showed scallop appearance and exhibited many stress concentration sites. In contrast, a significant E/H difference between (Cu,Ni)_6_Sn_5_ (18.45) and (Ni,Cu)_3_Sn_4_ (32.17) caused discontinuity in IMC mechanical behavior, thereby giving rise to cracking in-between those two phases and inferior impact resistance.

## 4. Conclusions

This study investigated impact properties of solder joints, nanomechanical behavior of IMCs at joint interface, and clarified the relationship between them. The IMCs at joint interface in the as-reflowed state were Cu_6_Sn_5_/Cu_3_Sn for Sn-Ag-Cu/Cu, Ag_3_Sn for Sn-Ag-Cu/Ag, (Cu,Ni)_6_Sn_5_/(Ni,Cu)_3_Sn_4_ for Sn-Ag-Cu/Ni, Ni_3_Sn_4_ for Sn-Ag/Ni and Cu_5_Zn_8_ for Sn-Ag-Zn/Cu. In combination with ball impact test results, it was found that the plastic ability of interfacial intermetallic compounds dominated impact force, impact toughness (impact energy) and the fracture mode of solder joints. Interfacial IMCs with a higher E/H exhibited an improved joint reliability, suffering high strain rate deformation and also ductile fracture. In addition, joint interfaces with bi-layered IMCs usually possessed inferior impact performance. The concepts of geometrical and mechanical discontinuities were applied to explain crack propagation paths for the bi-IMC layer interface.

## Figures and Tables

**Table 1:** Chemical compositions of the interfacial IMCs determined using energy dispersive spectroscopy (at%).

IMC/Composition	Sn	Ag	Cu	Ni	Zn
Cu_6_Sn_5_	44.2	---	55.3	---	---
(Cu,Ni)_6_Sn_5_	42.6	---	40.7	16.7	---
Ag_3_Sn	26.6	73.4	---	---	---
Ni_3_Sn_4_	59.9	---	---	40.2	---
Cu_5_Zn_8_	---	3.0	40.6	---	56.4

**Table 2:** Mechanical properties of the IMCs obtained using nanoindentation.

	H (GPa)	E (GPa)	E/H
Cu_6_Sn_5_	6.9 ± 0.22	130 ± 3.9	18.9
(Cu,Ni)_6_Sn_5_	7.0 ± 0.15	129 ± 1.3	18.5
Ag_3_Sn	1.9 ± 0.14	75 ± 2.6	39.5
Ni_3_Sn_4_	4.6 ± 0.19	148 ± 4.2	32.2
Cu_5_Zn_8_	7.0 ± 0.32	169 ± 3.3	24.1

## References

[1] Song J.M., Liu Y.R., Lai Y.S., Chiu Y.T., Lee N.C. (2012). Influence of trace alloying elements on the ball impact test reliability of SnAgCu solder joints. Microelectron. Reliab..

[2] Song J.M., Lin M.J., Hsieh K.H., Pai T.Y., Lai Y.S., Chiu Y.T. (2013). Ball impact reliability of Zn-Sn high temperature solder joints bonded with different substrates. J. Electron. Mater..

[3] Yang P.F., Lai Y.S., Jian S.R., Chen J., Chen R.S. (2007). Nanoindentation indentifications of mechanical properties of Cu_6_Sn_5_, Cu_3_Sn and Ni_3_Sn_4_ intermetallic compounds derived by diffusion couples. Mater. Sci. Eng. A.

[4] Deng X., Chawla N., Chawla K.K., Koopman M. (2004). Deformation behavior of (Cu,Ag)-Sn intermetallics by nanoindentation. Acta Mater..

[5] Chromik R.R., Wang D.N., Shugar A., Limata L., Notis M.R., Vinci R.P. (2005). Mechanical properties of intermetallic compounds in the Au-Sn system. J. Mater. Res..

[6] Lucas J.P., Rhee H., Subramanian K.N. (2003). Mechanical properties of intermetallic compounds associated with Pb-Free solder joints using nanoindentation. J. Electron. Mater..

[7] Song J.M., Liu Y.R., Su C.W., Lai Y.S., Chiu Y.T. (2008). Intermetallic formation induced substrate dissolution in electroless Ni(P)-solder interconnections. J. Mater. Res..

[8] Fisher-Cripps A.C. (2004). Nanoindentation.

[9] Song J.M., Shen Y.L., Su C.W., Lai Y.S., Chiu Y.T. (2009). Strain rate Dependence on Nanoindentation Responses of Interfacial Intermetallic Compounds in Electronic Solder Joints with Cu and Ag Substrates. Mater. Trans..

[10] Song J.M., Su C.W., Lai Y.S., Chiu Y.T. (2010). Time Dependent Deformation Behavior of Interfacial Intermetallic Compound Layers in Electronic Solder Joints. J. Mater. Res..

[11] Song J.M., Huang B.R., Liu C.Y., Lai Y.S., Chiu Y.T., Huang T.W. (2012). Nanomechanical Responses of Intermetallic Phase at the Solder Joint Interface—Crystal Orientation and Metallurgical Effects. Mater. Sci. Eng. A.

[12] Song J.M., Lu W.C., Chou P.W. (2020). Nanomechanical responses of intermetallic compound layer in transient liquid phase bonding using indium. J. Electron. Mater..

[13] Yu J.J., Wu J.Y., Yu L.J., Yang H.W., Kao C.R. (2017). Micromechanical Behavior of Single-Crystalline Cu6Sn5 by Picoindentation. J. Mater. Sci..

[14] Randle V., Powell G.L.F. (1997). The effect of Si on the relationship between orientation and carbide morphology in high chromium white iron. J. Mater. Sci..

[15] Oliver W.C., Pharr G.M. (1992). An improved technique for determining hardness and elastic modulus using load and displacement sensing indentation experiments. J. Mater. Res..

[16] Lai Y.S., Chang H.C., Yeh C.L. (2007). Evaluation of solder joints strengths under ball impact test. Microelectron. Reliab..

[17] Lai Y.S., Yang P.F., Yeh C.L. (2006). Experimental studies of board-level reliability of chip-scale packages subjected to JEDEC drop test condition. Microelectron. Reliab..

[18] Lai Y.S., Song J.M., Chang H.C., Chiu Y.T. (2008). Ball impact responses of Ni- or Ge-doped Sn-Ag-Cu solder joints. J. Electron. Mater..

[19] Yeh C.L., Lai Y.S., Chang H.C., Chen T.H. (2007). Empirical correlation between package-level ball impact test and board-level drop reliability. Microelectron. Reliab..

[20] Yang S.C., Chang C.C., Tsai M.H., Kao C.R. (2010). Effect of Cu concentration, solder volume, and temperature on the reaction between SnAgCu solders and Ni. J. Alloy. Comp..

[21] Ho C.E., Lin Y.L., Kao C.R. (2002). Strong effect of Cu concentration on the reaction between lead-free microelectronic solders and Ni. Chem. Mater..

